# Mendelian randomization analysis reveals a causal relationship between preterm birth and myopia risk

**DOI:** 10.3389/fped.2024.1404184

**Published:** 2024-07-18

**Authors:** Bin Lin, Long-long Chen, Dong-kan Li

**Affiliations:** ^1^ Xiamen Eye Center and Eye Institute of Xiamen University, Xiamen, China; ^2^ Xiamen Clinical Research Center for Eye Diseases, Xiamen, Fujian, China; ^3^ Xiamen Key Laboratory of Ophthalmology, Xiamen, Fujian, China; ^4^ Fujian Key Laboratory of Corneal & Ocular Surface Diseases, Xiamen, Fujian, China; ^5^ Xiamen Key Laboratory of Corneal & Ocular Surface Diseases, Xiamen, Fujian, China; ^6^ Translational Medicine Institute of Xiamen Eye Center of Xiamen University, Xiamen, Fujian, China

**Keywords:** Mendelian randomization, preterm birth, myopia, causal relationship, genome-wide association studies

## Abstract

**Background:**

Preterm birth has been associated with an increased risk of myopia, but the causal relationship between these two factors remains unclear. Traditional epidemiological studies are limited by confounding factors and reverse causality. Mendelian randomization (MR) analysis, utilizing genetic variants as instrumental variables, provides a robust approach to investigate causal relationships. In this study, we aimed to explore the potential causal link between preterm birth and myopia risk using a two-sample MR analysis strategy.

**Methods:**

We conducted a Mendelian randomization study to investigate the causal relationship between preterm birth and myopia risk. Genetic variants (single nucleotide polymorphisms, SNPs) were used as instrumental variables, and summary data from genome-wide association studies (GWAS) were utilized. Four regression models, including MR-Egger regression, weighted median regression, inverse variance weighted regression, and Weighted mode regression, were employed to validate the causal relationship. Sensitivity analysis was performed using the leave-one-out method. At the same time, the funnel diagram and MR-Egger test were used to judge the stability of the research results.

**Results:**

The MR analysis revealed a significant causal effect of preterm birth on myopia risk. Both the inverse variance weighted regression and weighted median regression models showed a *p*-value less than 0.05, indicating a robust association. The risk of myopia increased by approximately 30% for everyone standard deviation increase in preterm birth. Sensitivity analysis, funnel plot and MR-Egger test all confirm the stability of the research results.

**Conclusion:**

Our findings provide evidence supporting a causal relationship between preterm birth and myopia risk. Preterm infants are at a higher risk of developing myopia, and this association is not likely to be influenced by confounding factors or reverse causality. The SNP loci rs6699397, rs10871582, and rs2570497 should be closely monitored as they may lead to abnormal concentrations of intraocular cytokines, particularly vascular endothelial growth factor, potentially elucidating one of the pathogenic mechanisms contributing to the higher incidence of myopia in preterm infants. However the complex interconnections involved extend beyond these factors alone.

## 1 Background

With the rapid development of obstetrics and neonatal medical technology, the treatment of premature infants has made great progress, and an increasing number of diseases associated with premature infants have been identified. Among them, the development of myopia in premature infants has attracted much attention (1–3). According to statistics from the World Health Organization, about 153 million people suffer from unrecorded refractive errors every year (4), including children. There is no doubt that visual impairment has a significant impact on children's learning ability, and severe unrecognized refractive errors have a significant impact on children's development (5), leading to academic failure and impaired learning ability, and even affecting the future development of the country. The incidence of myopia in premature infants is significantly influenced by factors such as gestational age and birth weight, but currently, there is no conclusive evidence indicating that prematurity is a cause of myopia (6).

Prematurity is increasingly recognized as an important risk factor for the development of myopia (7), although conclusive evidence is still lacking. Myopia is a common refractive error characterized by elongation of the eyeball, which causes light to focus in front of the retina. Its etiology is multifactorial, involving genetic and environmental influences (8). Recent advances in genetic epidemiology have elucidated many genetic variations associated with myopia (9). However, the interaction between genetic myopia and prematurity is still poorly understood. This knowledge gap emphasizes the need for comprehensive research to elucidate the genetic link between prematurity and subsequent myopia risk. Understanding these relationships is crucial for the development of prevention strategies and therapeutic interventions aimed at reducing the risk of myopia in the population at risk for prematurity.

In traditional epidemiological studies, the association between exposure factors and health outcomes is often confounded by unmeasured confounding factors and reverse causality, which limits the accuracy of causal inference. Mendelian randomization (MR) method, as a causal inference tool based on genome-wide association study (GWAS) data, has been widely used in recent years (10). The MR method utilizes the random distribution characteristics of genetic variations during gamete formation, and theoretically avoids the influence of confounding factors in traditional observational studies (11). At the same time, because the genetic variations explained by these genetic variations precede the occurrence of health outcomes in time, the MR method also helps to exclude the possibility of reverse causality (12).

**Figure 1:**
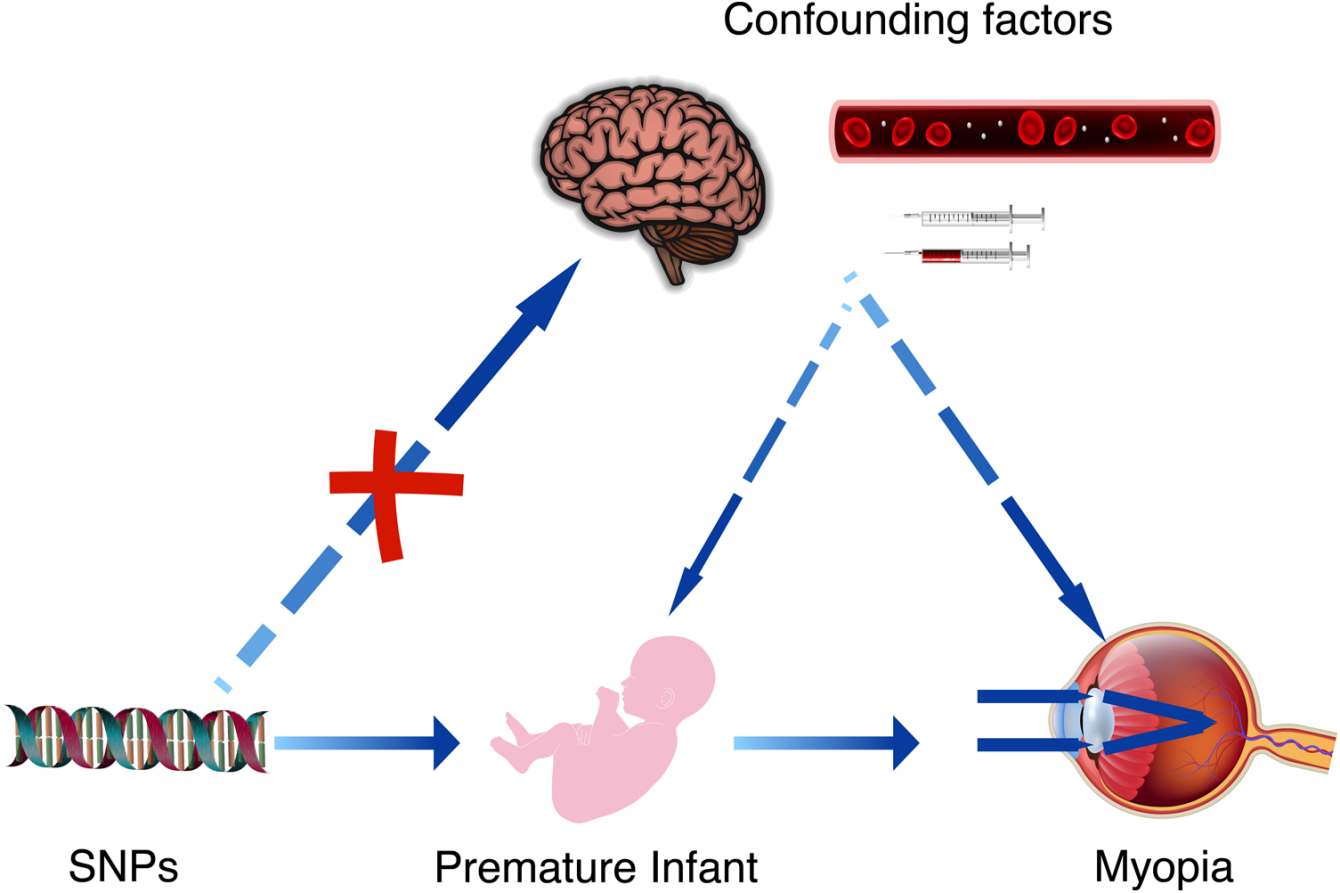
The causal relationship between premature infant and myopia can be further confirmed by Mendelian randomization studies and the effect of confounding factors can be excluded. These confounding factors may include brain damage caused by hypoxia in premature infants, as well as the complications arising from related treatments.

In this study, we used the so-called two-sample MR analysis strategy, using single nucleotide polymorphisms (SNPs) as instrumental variables, based on summary data from GWAS to investigate the potential causal relationship between prematurity and myopia. Through this gene-level analysis, we aim to overcome the limitations of traditional research methods and provide more reliable evidence to support the causal relationship between prematurity and myopia. As shown in Figure 1.

## 2 Method

We conducted an MR study to investigate the causal relationship between preterm birth and the risk of myopia. The MR method uses genetic variation as an instrumental variable to estimate the causal effect of exposure (preterm birth) on the outcome (myopia risk) in the presence of confounding factors. All statistical analyses were performed using the R programming language, specifically using software packages designed for MR studies such as TwoSampleMR and Mendelian Randomization.

### 2.1 Data source

We obtained GWAS data for the largest sample size of Preterm Birth (Pubmed ID: 34140656) and Myopia (Pubmed ID: 34140656) from the Social Science Genetic Association Consortium (SSGAC) website (https://www.thessgac.org). The website was accessed on March 10, 2024. The population sources for both datasets were European, with no gender restrictions. The number of SNPs in the preterm birth dataset was 2,001,934, and the number of SNPs in the myopia dataset was 6,485,931.

### 2.2 Condition setting

Instrumental Variable Conditions: The conditions for selecting SNPs as instrumental variables were as follows: ① The instrumental variables were highly correlated with the exposure, with an F = beta^2^/se^2 ^> 10 as a strong correlation criterion (13, 14). ② In the study, a test for genetic pleiotropy was conducted, with a *P*-value of ≥0.05 indicating no genetic pleiotropy. This suggests that the instrumental variables affected the outcome solely through the exposure without any direct link. To clarify further, if the results of the genetic pleiotropy test had shown a *P*-value < 0.05, it would imply the presence of some inextricable association between the variables that could not be eliminated (15). ③ The instrumental variables were unrelated to unmeasured confounding factors. Since the SNPs selected by the MR method follow the genetic principle of random allocation of alleles from parents to offspring, the influence of environmental and postnatal factors is minimal. Therefore, it can be theoretically assumed that the instrumental variables are independent of environmental factors such as socioeconomic and cultural factors.

### 2.3 SNP selection

Given the substantial size of our original GWAS dataset, meaningful SNPs were selected from the GWAS summary data of preterm birth based on a screening criterion of *P* < 5 × 10^−8^ rather than *P* < 5 × 10^−7^ nor *P* < 5 × 10^−6^, to ensures that the SNPs selected have a very significant association with the traits under study.

If a SNP strongly associated with a disease is in linkage disequilibrium with another SNP that does not directly cause the disease, the latter may also serve as a marker for research and diagnosis. To avoid this, the linkage disequilibrium coefficient (*r*^2^) was set to 0.001. Because of the extremely low *r*^2^ value, to avoid overlooking distal associations, the linkage disequilibrium region width was set to 10,000 kb to ensure the independence of each SNP and exclude the influence of genetic pleiotropy (16, 17).

The preterm birth-related SNPs were extracted from the myopia GWAS summary data, with a minimum *r*^2 ^> 0.8 set to ensure the accuracy of the results. Missing SNPs were directly deleted. The information from the two datasets was combined, and SNPs directly related to myopia (*P* < 5 × 10^−8^) were removed (18).

### 2.4 Causal relationship verification

Four regression models, MR-Egger regression, weighted median estimator (WME), inverse-variance weighted (IVW) random-effects model, and Weighted model, were used to verify the causal relationship between exposure (preterm birth) and outcome (myopia) using SNPs as instrumental variables. The IVW method does not require individual-level data and can directly calculate the causal effect estimates using summary data. MR-Egger regression fits a linear function by calculating the correlation between each SNP and myopia (Y) and the correlation between each SNP and preterm birth (X). The WME method calculates the causal effect estimate (βj) of the exposure outcome for the jth SNP. MR-Egger formula was applied to assess heterogeneity among SNPs. If heterogeneity exists, the IVW method using robust regression with penalized weights may be a worthwhile additional sensitivity analysis to be performed in a Mendelian randomization analysis (19). Sensitivity analysis was performed using the leave-one-out method. If the results remain stable after removing any particular SNP, this indicates that our results are less dependent on a single genetic variable with a strong pleiotropy. The symmetry of the funnel plot also plays a crucial role; if the funnel plot exhibits a uniform and symmetric distribution of results, it suggests a low likelihood of publication bias or other systematic errors. Even in the presence of pleiotropy, a symmetric funnel plot indicates that, although pleiotropy exists, it may not significantly bias the results in any particular direction, or its impact is consistent across different studies. It is important to note that if the y-intercept of the MR-Egger regression is less than 0.05, it generally indicates the absence of significant directional pleiotropy, meaning there is no evidence that the instrumental variables systematically affect the outcome variable through unobserved pathways.

And the parameters for creating the heatmap are as follows: SNP identifiers are used as the horizontal axis, while the effect sizes of the outcome variable, denoted as BETA values, are used as the vertical axis. The standard error of the outcome variable, referred to as SE values, is used for the fill. This heatmap not only displays the effect sizes of each SNP but also visualizes the statistical confidence of these effect sizes through variations in color intensity. All of the above methods were implemented using the TwoSampleMR 0.5.10 package in RStudio 4.3.3 software, with a significance level of *α* = 0.05.

## 3 Results

### 3.1 SNP information screening results

A total of 2,001,934 SNP information was obtained for preterm birth. After filtering based on a criterion of Pval < 5 × 10^−8^, 1,017 SNPs remained. The file was exported and placed in the TwoSampleMR folder. After renaming the sequence names, SNPs were selected to ensure independence by setting a linkage disequilibrium coefficient (*r*^2^) of 0.001 and a linkage disequilibrium region width of 10,000 kb, excluding the influence of genetic pleiotropy. This resulted in the removal of 973 SNPs, leaving 44 SNP data. Upon calculating the F-statistics for a selected set of 44 SNPs, it was found that all 44 SNPs had F-values greater than 10, with an average F-value of 41.28. This indicates that the reliability and strength of these 44 SNPs as instrumental variables are considerably high. The final intersection of the selected preterm birth and myopia data yielded 44 SNPs. No outliers were found after sensitivity analysis, and these 44 SNPs were included. The basic information of the SNPs is shown in Table 1.

**Table 1:** Summary of the selected SNP information.

SNP	CHR	BP	A1	EAF	BETA	SE	F
rs10161336	12	97673417	G	0.52142	−0.00575	0.00364	2.495358
rs10496880	2	142237435	T	0.93408	0.00663	0.00726	0.833977
rs10510027	10	118869607	C	0.20226	0.00265	0.00481	0.30353
rs10810099	9	14161927	A	0.29824	−0.00265	0.00408	0.421863
rs10871582	18	53276589	G	0.66534	0.01356	0.00384	12.46973
rs11192193	10	106569253	A	0.40189	−0.01297	0.00371	12.22171
rs11581644	1	153890988	A	0.69686	0.00971	0.00399	5.922331
rs11664298	18	77578986	G	0.81429	0.00237	0.00449	0.278615
rs11709466	3	84610654	T	0.59038	0.00022	0.0037	0.003535
rs11743711	5	60735530	T	0.61441	0.0059	0.00373	2.501995
rs11762636	7	2061111	C	0.79205	−0.01442	0.00471	9.373218
rs12220267	10	105075712	C	0.65793	−0.0057	0.00391	2.125182
rs12514615	5	45253659	A	0.81748	−0.00506	0.00479	1.115912
rs12588538	14	103360000	A	0.8219	−0.00276	0.00488	0.319874
rs13013603	2	49718140	C	0.66226	−0.00247	0.00388	0.405257
rs13245564	7	136989304	G	0.55621	−0.00461	0.0037	1.552381
rs1372171	8	87690145	G	0.15627	0.00757	0.00481	2.476861
rs1606183	12	84011789	A	0.65982	−0.00488	0.00378	1.666695
rs1701704	12	56412487	T	0.68334	−0.00771	0.00383	4.05239
rs1941954	18	35159596	A	0.33536	−0.01045	0.00389	7.216612
rs1947114	2	166180772	A	0.73879	−0.00817	0.00415	3.87568
rs2160515	12	16753965	A	0.56971	−0.00564	0.0037	2.323565
rs2347867	6	152229850	G	0.36746	−0.00867	0.00377	5.28878
rs2570497	2	104441546	C	0.35579	0.01273	0.00379	11.2818
rs2777888	3	49898000	A	0.51663	0.00773	0.00365	4.485112
rs2871304	4	27983557	G	0.25254	−0.00596	0.00409	2.123469
rs293566	20	31097877	T	0.62321	0.0066	0.00388	2.893506
rs359233	2	60470926	A	0.35989	0.00777	0.00378	4.225309
rs3769184	2	174019920	A	0.40577	−0.00445	0.00377	1.393277
rs4438499	2	100876789	G	0.51323	−0.00577	0.00367	2.471835
rs4814324	20	14742830	C	0.49838	−0.00111	0.00367	0.091477
rs587508	1	229980610	G	0.48201	0.00139	0.00364	0.145823
rs6137217	20	20997702	C	0.66675	−0.00449	0.00388	1.33915
rs6511036	19	19582651	A	0.82886	−0.00618	0.00477	1.678573
rs6699397	1	91212216	A	0.6033	0.01459	0.00377	14.97711
rs6980093	7	114162740	G	0.42529	0.00193	0.00374	0.2663
rs7024505	9	14712257	A	0.60816	0.00099	0.00379	0.068233
rs7324673	13	67145057	A	0.85761	0.00063	0.00534	0.013919
rs750472	8	145701453	A	0.52053	−0.00141	0.00364	0.15005
rs769669	4	140884193	C	0.68323	−0.00641	0.00388	2.729308
rs9267576	6	31812038	T	0.11769	0.00734	0.00509	2.079489
rs9352357	6	63689527	C	0.68933	0.00037	0.00393	0.008864
rs9372625	6	98344031	G	0.63326	−0.01136	0.00375	9.17686
rs9561317	13	93990654	C	0.85872	−0.00825	0.00542	2.316911

SNP, SNP number; CHR, chromosome number; BP, location; A1, effector allele; EAF, effect allele frequency.

### 3.2 Heterogeneity test

The MR-Egger regression results showed a statistic Q = 78.65 (*P* < 0.001), indicating the presence of heterogeneity among the SNPs. Therefore, the focus should be on the random-effects IVW model.

### 3.3 Causal relationship verification

**Figure 2:**
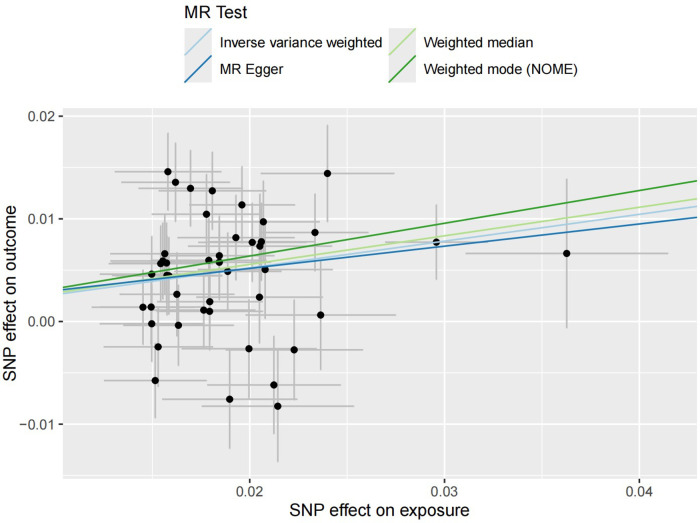
Four scatter plots of regression models are shown in the figure. The intersection of the four regression lines near the origin suggests the presence of very slight confounding factors in this study. Additionally, the slope of the regression lines minus one represents the increased risk of disease for every standard deviation increase in the risk factor.

**Figure 3:**
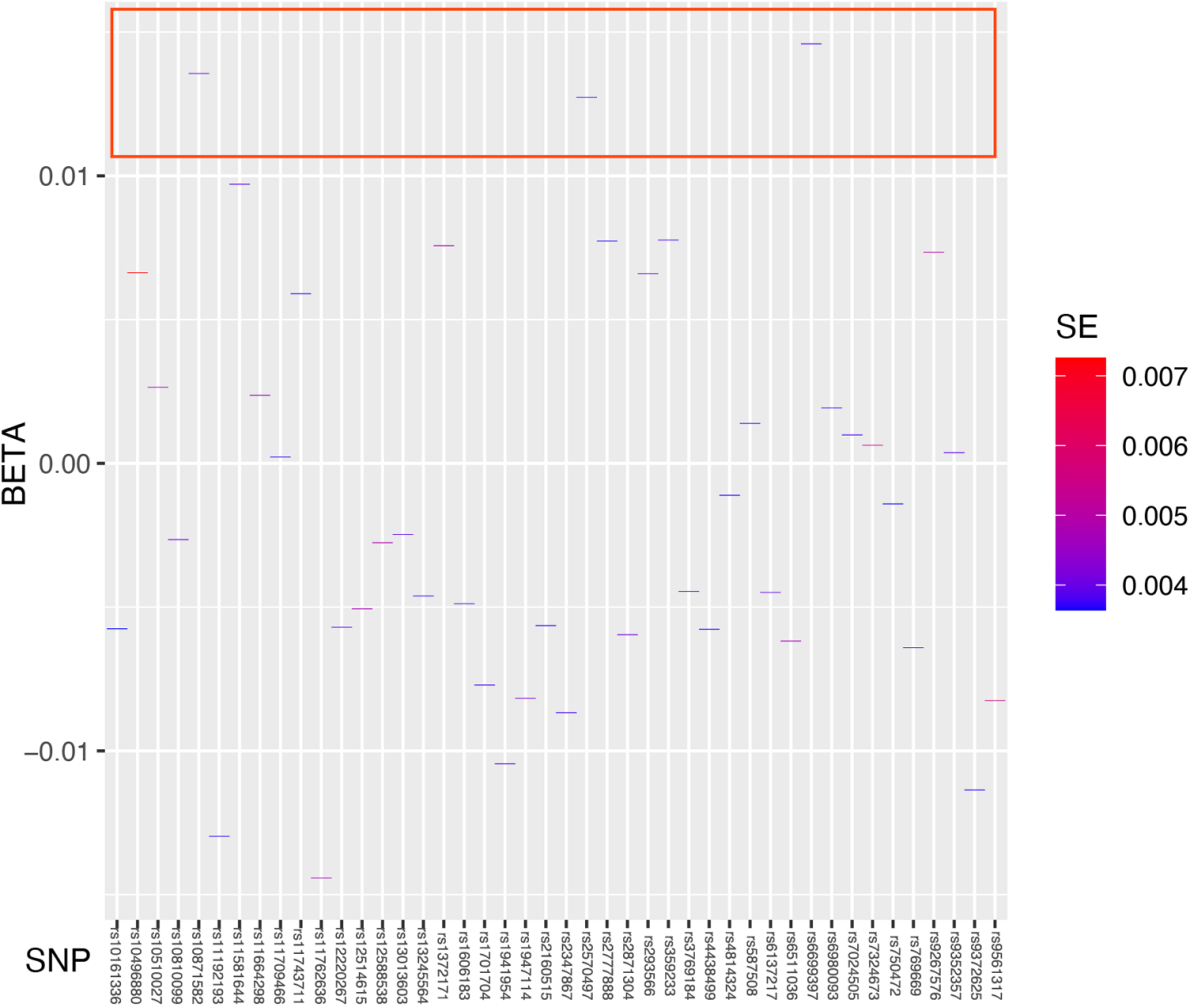
Heatmap showing the results of Mendelian randomization analysis. We used Standard Error as the fill data, where lower values are indicated by blue and higher values by red. The blue coloration signifies lower standard errors, suggesting that the estimates of the effect size of the exposed variable are more precise and reliable. The deep blue color indicates that the effect size estimates for specific SNPs such as rs6699397, rs10871582, and rs2570497 are highly trustworthy with a high statistical confidence level. These SNPs which are inside the red box, positioned at the top of the heatmap, exhibit the highest credibility and thus warrant focused research and discussion.

The regression results of the four methods are shown in Table 2. Both the inverse variance weighted regression model and the weighted median regression model yielded *P* < 0.05. Based on the results of the heterogeneity test, the main focus should be on the IVW regression model, which suggests that preterm birth is a risk factor for myopia in newborns. Additionally, for every increase of 1 standard deviation, the risk of myopia increases by approximately 30%. The scatter plot is shown in Figure 2. The four regression lines in the graph do not pass through the coordinate origin, often interpreted as evidence of genetic confounding or invalid instrumental variables, particularly in the MR-Egger regression line. However, their intercepts with the y-axis are significantly less than 0.01, much lower than the commonly used threshold of 0.05 (20, 21). Indicating minimal interference factors. We make the heatmap by the strategy mentioned in the Method, and the display is illustrated as shown in the Figure 3. As observed, the presence of the SNP loci rs6699397, rs10871582, and rs2570497 is associated with a positive effect on the exposure variable. Specifically, an increase in the phenotype associated with these SNPs, which influences preterm birth, indirectly elevates the risk of myopia.

**Table 2:** Regression model results of the four methods.

Method	*β*	se	OR(95% CI)	*p*
MR-Egger	0.216	0.239	1.242 (0.776–1.986)	0.372
WME	0.278	0.052	1.321 (1.192–1.463)	<0.05
IVW	0.261	0.044	1.300 (1.191–1.415)	<0.05
Weighted mode	0.323	0.107	1.381 (1.120–1.703)	<0.05

WME, weighted median estimator; IVW, inverse-variance weighted.

### 3.4 Sensitivity analysis

**Figure 4:**
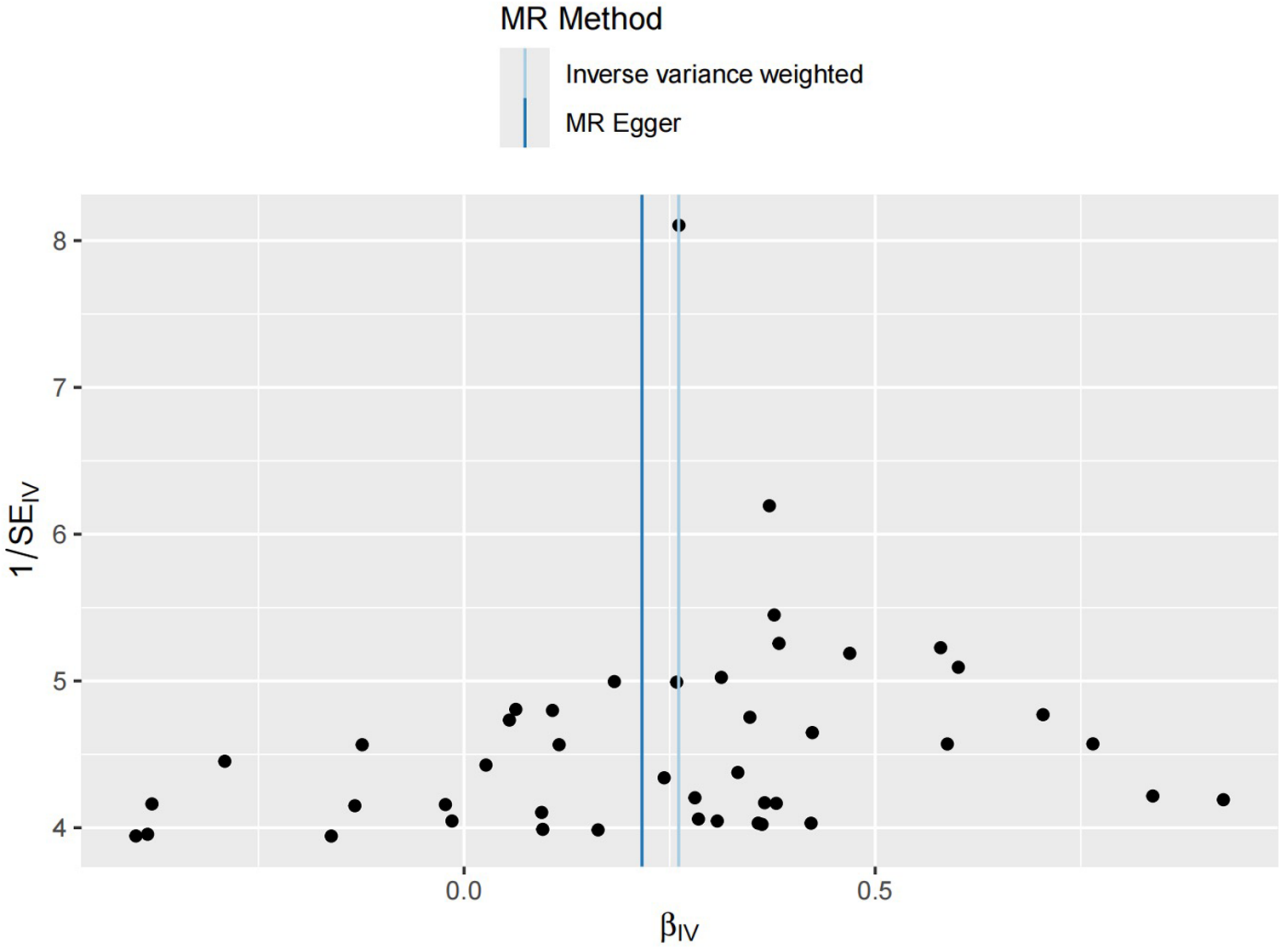
Funnel plot distribution of 44 SNP information. The funnel plot symmetry generally indicates an absence of significant genetic heterogeneity or measurement bias. The symmetric central axis in the MR-Egger method suggests that the results are reliable and free from significant genetic confounding. The IVW method assumes that all instrumental variables are valid, indicating no genetic confounding. The conclusions drawn from both methods are broadly similar, further validating the reliability of this study's conclusions.

**Figure 5:**
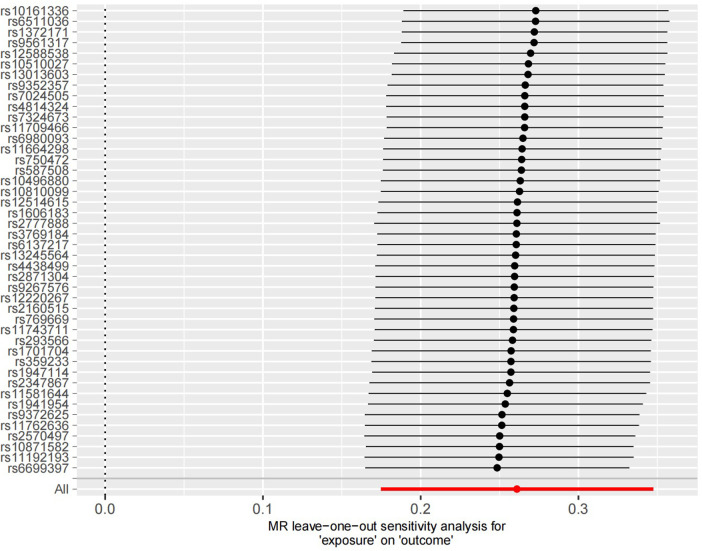
Sensitivity analysis results of the 44 SNP information. It can be observed that all statistical results are positioned on the right side of the vertical axis, demonstrating good stability.

The sensitivity analysis was performed using the leave-one-out method, and the results showed that regardless of which SNP was removed, the remaining SNPs consistently fell to the right of the null line, indicating that the effect estimates remained positive and close to the overall effect estimate. This suggests that removing any individual SNP would not have a significant impact on the results, indicating the robustness of the MR findings in this study. The funnel plot and detailed sensitivity analysis results can be found in Figures 4, 5, respectively.

## 4 Discussions

According to reports, premature infants are more prone to develop myopia. Larsson and Holmstrom (22) found through a 10-year follow-up study that premature infants have a significantly higher risk of visual impairment and significant refractive errors compared to full-term infants.

Previous studies have suggested that the refractive status of premature infants may be related to gestational age, birth weight, head size, and body length (23–25). In exploring the relationship between weight and myopia, Modrzejewska et al. (26) found that infants with birth weights between 1,556 g and 1,621 g were more likely to be hyperopic at 64 weeks post-term, while infants with birth weights between 810 g and 1,234 g were more likely to be myopic. Several long-term studies have also supported this view (27, 28), suggesting that low birth weight is a risk factor for myopia development. In contrast, Ton et al. (29) reported that gestational age and birth weight had no effect on the refractive status of premature infants. Therefore, D. Plotnikov et al. (30) found through Mendelian randomization study that lower birth weight within the normal range is causally associated with more severe myopic refractive errors, but the effect of this causal relationship is weak.

Based on the above, this study further confirms the causal relationship between gestational age of preterm infants and myopia in the European population through Mendelian randomization. In our Mendelian randomization study, we found that for each standard deviation increase in the exposure factor (prematurity), the risk of myopia increased by 30%, and this conclusion was stable upon examination. Additionally, heatmap analysis identified three SNPs—rs6699397, rs10871582, and rs2570497—as exerting the most significant positive effects on the outcome variable.

The results of the heterogeneity test (*P* < 0.001) also indicate that there may be some confounding factors between the two variables we studied that we were unable to eliminate, such as the retinopathy of prematurity (ROP). Therefore, employing methods such as funnel plots, leave-one-out tests, and MR-Egger regression to assess and mitigate these potential biases is crucial. Each method offers unique insights, as detailed in the Section 1.4 Causal Relationship Verification. As demonstrated in our results, the intercept of MR-Egger regression approaches zero, the funnel plot exhibits symmetric data distribution, and leave-one-out tests indicate weak dependency on individual variables. These findings collectively support the accuracy and validity of the study results. So, we proceed with the following discussion for the results.

In ophthalmology, ROP is a common disease in premature infants (31) and the leading cause of blindness in preschool children (32). Previous studies (33, 34) have shown that full-term infants often have a certain degree of hyperopic reserve, while in non-ROP premature infants, only 266 out of 469 cases (56.72%) had hyperopic refractive status. In premature infants with ROP, this probability decreased to 39 out of 86 cases (45.35%), lower than the probability of myopic refractive status, which is 42 out of 86 cases (48.84%). It can be seen that ROP also plays an important role in the onset of myopia. However, this also suggests that ROP in preterm infants could potentially serve as a confounding factor in this study.

To explore potential links between the three SNPs and the onset of ROP and myopia, we conducted a literature review. We have discovered that abnormal expression in the Chromosome 11q (35, 36) may increase the risk of Retinopathy of Prematurity in neonates (37). Detailed gene information obtained from the Ensembl website (http://asia.ensembl.org/index.html) revealed that rs6699397 is located at 1p22.2, rs2570497 at 2q12.1, and rs10871582 at 18q21.2, with no significant link with the Chromosome 11q. This appears consistent with our study findings, suggesting that there are no significant confounding factors between preterm birth and myopia.

Further literature searches on these chromosomal regions led to the surprising discovery that they are all associated with the development of intraocular neovascularization (38–40). It is well known that neovascularization plays an important role in the pathogenesis of ROP. However, upon careful examination of the three related literature and considering the results of the confounding analysis from this study, we believe that the two mechanisms are different in causing neovascularization. Additionally, research by Qiaoling Wei et al. (41) in 2021 suggested that the development of myopia may be linked to elevated intraocular Vascular Endothelial Growth Factor (VEGF) levels. Thus, we hypothesize that one mechanism by which the risk of myopia increases in premature infants could be due to specific genetic loci altering the concentration of certain cytokines within the eye, thereby promoting the onset of myopia. Facing results indicating no significant confounding factors, yet observing intricate connections among preterm birth, VEGF, and myopia, we infer that the formation of neovascularization within the eye might be a component in the causal mechanism whereby preterm birth leads to myopia. Assuming A represents preterm birth, A1 represents VEGF, B represents ROP, and C represents the risk of myopia, we can summarize the relationship as follows: A leads to A1, which then leads to C, rather than a confounding relationship where A directly causes C while also causing B to lead to C. This could potentially explain the results of Mendelian randomization analysis and confusion analysis.

While this study primarily explores the genetic association between preterm birth, VEGF, ROP, and myopia in a European population, its findings also hold implications for early myopia prevention strategies in other populations or countries. Looking ahead, as we have to confront challenges like preterm birth, these SNPs, highlighted in the heatmap as high-risk, could potentially serve as novel targets for therapeutic intervention. However, genetic expression is a vast and intricate field of study, and we acknowledge that our investigation represents merely a fraction of the theoretical landscape. The ultimate accuracy of these conclusions requires further clinical validation.

## 5 Conclusions

Based on the results of our study, we may draw the following conclusions regarding the potential causal relationship between preterm birth and myopia risk: Preterm birth is associated with an increased risk of myopia. Our Mendelian randomization analysis revealed a significant causal relationship between preterm birth and myopia risk. For every increase of one standard deviation in prematurity, the risk of myopia increases by approximately 30%. This study suggests that preterm birth should be considered a risk factor for myopia in newborns. Furthermore, it was found that the abnormal expression of SNP loci rs6699397, rs10871582, and rs2570497 may lead to abnormal concentrations of intraocular cytokines, particularly vascular endothelial growth factor, potentially elucidating one of the pathogenic mechanisms contributing to the higher incidence of myopia in preterm infants. However, this study ultimately focused solely on the network relationships between preterm birth, VEGF, ROP, and myopia. We believe that the complex interconnections involved extend beyond these factors alone. We hope that these findings will provide assistance and insights for future observations of vision in preterm infants.

## Data Availability

The original contributions presented in the study are included in the article/Supplementary Material, further inquiries can be directed to the corresponding author.
